# The utility of routine cultures, cell count, and crystal evaluation of aspirate from aseptic olecranon bursitis

**DOI:** 10.1016/j.jseint.2022.02.012

**Published:** 2022-03-18

**Authors:** Sebastian Bustamante, Michael Boin, John Dankert, David Adekanye, Mandeep S. Virk

**Affiliations:** Department of Orthopedic Surgery, New York University Langone Orthopedic Hospital, New York, NY, USA

**Keywords:** Elbow, Diagnostics, Olecranon bursitis, Bursitis, Olecranon, Aspiration, Elbow trauma

## Abstract

**Background:**

Aspiration of the olecranon bursa is a treatment option for acute olecranon bursitis (OB). Typically, the aspirate is sent for microbiologic analysis, cell count, and crystal analysis. This study investigates the utility of fluid aspirate analysis from patients with clinically diagnosed aseptic OB.

**Methods:**

In this prospective study (IRB #i20-00986), patients presenting with acute aseptic OB were treated with aspiration as standard of care. Patients consented to participate in this study via phone. Patients with suspected infectious bursitis, open draining wound, and chronic OB were excluded. The aspirate was sent out for routine microbiologic analysis (aerobic and anaerobic cultures and Gram staining) and fluid analyses, including cell count with differential and crystal analysis. Nucleated and differential cell count was reported as absolute numbers per cubic millimeter and percentage, respectively. Compression wrap was applied after OB aspiration, and patients were asked to ice and take anti-inflammatory medications. Clinical follow-up was done after 6 weeks and at 3 months for resolution vs. recurrence of symptoms, and the mean time to resolution was reported.

**Results:**

A total of 26 patients (28 cases) with aseptic OB were enrolled in this study. Two patients had bilateral OB. The mean time to aspiration after the onset of symptoms was 26.4 days. One patient had recurrence of swelling after the first aspiration and underwent repeat bursa aspiration. No organisms were isolated or reported on Gram staining on any of the aspirate samples. Two aspirates were reported positive for calcium pyrophosphate dihydrate crystals. No patient had monosodium urate crystals. All patients had resolution of swelling and symptoms without the development of postaspiration infection.

**Conclusions:**

This study demonstrates limited clinical utility of routine microbiologic analysis (cell count, microbiologic, and crystal evaluation) of fluid aspirate from clinically diagnosed aseptic OB. Although 7% of fluid aspirates were positive for calcium pyrophosphate dihydrate crystals, it did not change the overall treatment.

Olecranon bursitis (OB) can be either septic or aseptic, with considerable overlap between their history and clinical presentation.^2^, ^3^, ^4^, ^5^^,^^7^ It has been reported that OB is typically noninfectious in origin, with septic cases comprising only 20%-33% of diagnosis.^1^^,^^3^^,^^9^ Both types usually present as an elbow swelling with pain being less common in aseptic OB unless associated with considerable trauma.^1^^,^^2^^,^^4^^,^^5^ Septic OB typically has overlying erythema extending beyond the olecranon bursal region, warmth to touch, elbow pain and in some cases can have a draining sinus or purulent drainage.^2^^,^^6^^,^^8^^,^^9^ Differentiating between these 2 categories is important because their treatments are different.^1^

Regardless of the type, the initial diagnosis of OB is based on the clinical presentation, patient history, and physical examination.^1^^,^^5^^,^^8^ There is no general consensus on how to treat these patients, and so it tends to vary based on the physician’s preference as well as the patient’s own personal preferences.^4^^,^^10^ Treatment options include oral anti-inflammatory medications, topical ice application, bursal aspiration with or without steroid injection, and, in rare chronic cases, even surgery.^4^, ^5^, ^6^, ^7^, ^8^, ^9^, ^10^

Aspiration can be both therapeutic and diagnostic. The therapeutic benefits include reduction of swelling size and discomfort.^4^^,^^6^^,^^8^ Typically, it is recommended to send aspirate for microscopic analysis (cell count, microbiologic, and crystal analysis).^1^^,^^4^, ^5^, ^6^^,^^9^^,^^11^ However, the utility of routine fluid analysis of an OB aspirate in clinically determined aseptic OB is not known. The aim of this prospective study was to determine the utility of routine microscopic fluid analysis of OB aspirate in patients with clinically determined aseptic OB. Our hypothesis was that there would be no change in the treatment plan with microscopic analysis (cell count, microbiologic, and crystal analysis) of the aspirated OB fluid, and therefore, microscopic analysis of bursal fluid is not necessary in clinically diagnosed aseptic OB.

## Materials and methods

### Study design

From August 2020 to July 2021, 26 patients with OB were treated with aspiration of their olecranon bursa at our center. One patient was treated during the same visit for bilateral OB, and another had both elbows treated but each elbow on separate visits. This totals 28 different olecranon bursae aspirated during this time. Eight patients elected to have their OB treated without aspiration during the same time. All patients underwent plain radiographs of the affected elbow.

None of the patients reported fever, cough, dysuria, significant pain, or other symptoms suggestive of septic OB. Exclusion criteria were drainage from bursa and chronic OB. We defined chronic OB as swelling persisting for more than 3 months.

### Treatment

The senior author performed all aspirations using sterile technique without ultrasound guidance. The aspirated fluid was characterized as being serous (straw color), purely hemorrhagic, or mixed based on its external appearance. The aspirate was sent for routine microbiologic analysis (aerobic and anaerobic cultures and Gram staining) and fluid analyses including cell count with differential and crystal analysis. Nucleated and differential cell count was reported as absolute numbers per cubic millimeter and percentage, respectively. Crystal analysis included the presence or absence of calcium pyrophosphate dihydrate (CPPD) or monosodium urate crystals (MSU).

After aspiration, a compression wrap was applied over the elbow and held in place for 24-48 hours. All patients were instructed to apply ice topically for 15 minutes every 2-3 hours when awake and take oral anti-inflammatory medications for 7 days (ibuprofen 600 mg TID). Patients were instructed to report any erythema, drainage, or recurrence of swelling during the follow-up period. Clinical follow-up was done after 6 weeks and at 3 months for resolution vs. recurrence of symptoms, and the mean time to resolution was noted.

## Results

### Clinical characteristics

Of the 26 patients, 18 were male and 8 were female. The average age was 59 years (range, 33-90 years). Twelve of the aspirated bursae were on the patient’s dominant arm, and 16 were on the nondominant arm. Twenty-one cases were on patients who reported no inciting trauma to the elbow before symptoms appeared. One patient reported that he had been skiing before both of his elbows started swelling up. Five patients reported direct trauma to the elbow: 3 from hitting the elbow against a hard surface and 2 from a fall onto the elbow. Twenty-six of the cases had never been aspirated before; one was aspirated after failed initial conservative treatment at an outside hospital. One OB case had already been aspirated at least one time before receiving treatment at our center. The average time from symptom appearance to aspiration was 26.4 days (range, 3-90 days). None of the patients had a history of hyperuricemia, but 3 patients were positive for at least 3 of the diagnostic criteria for metabolic syndrome, indicating a positive diagnosis for metabolic syndrome (Table I). Eight aspirates were characterized as serous in nature, 3 were characterized as hemorrhagic, and 17 were characterized as a mix of blood and serous fluid.

**Table I:** Patient demographics and comorbidities.

Total patients	26
Total cases	28
Sex, n (%)	
Male	18 (69.23)
Female	8 (30.77)
Age, mean (range)	59 (33–90)
Dominant hand, n (%)	
Right	25 (96.2)
Left	1 (3.8)
Laterality, n (%)	
Right	11 (39.29)
Left	17 (60.71)
Mechanism of injury, n (%)	
Atraumatic	21 (75)
Traumatic	7 (25)
Time between onset of symptoms and aspiration, mean, days, (range)	26.4 (3–90)
Comorbidities (n)	Anemia (1) Anterior uveitis and iritis (1) Arrythmia (3) Asthma (4) Benign prostatic hyperplasia (3) Breast cancer (1) Choroiditis (1) Chronic kidney disease (2) Clotting disorder (2) Coronary artery disease (4) COPD (1) Depression (2) Diabetes (2) Deep vein thrombosis (2) Epilepsy (1) GERD (5) Glaucoma (2) Hepatitis C (1) Hypercholesterolemia (1) Hyperlipidemia (16) Hypertension (10) Hyperuricemia (0) Hypothyroidism (3) Inflammatory bowel disease (1) Lateral epicondylitis (1) Lyme disease (1) Melanoma (1) Metabolic syndrome (3) Migraines (2) Nephrolithiasis (1) Osteoarthritis (2) Osteoporosis (4) Paget-Schrotter syndrome (1) Parkinson disease (1) Peripheral vascular disease (1) Prostate cancer (1) Rheumatoid arthritis (1) Seizures (1) Squamous cell carcinoma (1) TIA (1) Varicose veins (1)

*COPD*, chronic obstructive pulmonary disease; *GERD*, gastroesophageal reflux disease; *TIA*, transient ischemic attack.

### Microbiologic analysis

No organism was isolated or reported on Gram staining on any of the samples from the aspirates, and no bacterial growth was found on any of the cultures (Table II). The specimens were held for 14 days.

**Table II:** Microscopic analysis of fluid aspirate from olecranon bursitis in the study group.

Microscopic analysis parameter	Findings
Cell count, mean (range)	1289.5 (44–15,070)
Microbiological culture, n (%)	
Positive	0 (0)
Negative	28 (100)
Crystals, n (%)	
Monosodium urate	0 (0)
Calcium pyrophosphate dihydrate	2 (7.14)

### Cell count and crystal analysis

The average white cell count was 1289.5 cells/mm^3^ (range, 44-15,070 cells/mm^3^). Two aspirates reported positive for CPPD crystals. No patient had MSU crystals (Table II).

## Discussion

In this study, we found no utility of microbiologic analysis and crystal analysis of clinically determined aseptic OB. Two aspirates were positive for CPPD crystals, but this result did not change the treatment plan. None of the patients had positive cultures or Gram straining. All patients had resolution of their swelling.

The treatment of acute aseptic OB varies according to the patient and physician preferences, but typically one of the common decision-making includes whether to aspirate the bursa sac or not. Although differentiating between septic and nonseptic bursitis can be done clinically, it is not always easy to distinguish between the two.^1^^,^^2^^,^^10^ One of the approaches in making a definite diagnosis is to aspirate the OB and submit the fluid for microbiologic analysis and crystal analysis.^1^^,^^2^^,^^7^ This, however, is not a universal practice. Proponents of fluid aspirate analysis use this strategy to confirm the aseptic nature of the bursitis and to detect crystal-induced bursitis (gout or pseudogout). However, there is no evidence for or against routine microbiologic analysis and crystal analysis of clinically determined aseptic OB. Furthermore, the reliability of the cutoff laboratory values obtained on fluid analysis to distinguishing septic from aseptic OB has come into question.^7^ Truong et al have suggested high variability in the sensitivity of Gram staining, finding sensitivity values between 15% and 100%, and results being negative in half of all cases of septic bursitis,^12^ whereas Reilly et al suggested that positive Gram stains are found in 50%-100% of septic OB cases proven by positive cultures.^9^ These authors also found aspirate white blood cell counts to be unreliable in making this distinction- reporting counts between 690 cells/mm^3^ and 418,000 cells/mm^3^ in septic OB and between 50 cells/mm^3^ and 10,000 cells/mm^3^ in aseptic cases.^9^ In this study, we clinically characterized OB as aseptic if there was no erythema and/or drainage. To provide a meaningful value to this study, we excluded patients of OB with erythema or drainage or history of chronic OB.

In this study, we found that routine microbiologic analysis and crystal analysis did not change the treatment plan in clinically determined aseptic OB. This finding has important conclusions with respect to the treatment of OB. First, physicians who want to treat clinically determined aseptic OB without aspiration could do so with minimal risk of missing any finding that would change the treatment plan. Second, if fluid aspiration of an aseptic OB is performed, microbiologic or crystal analysis has a low diagnostic and or therapeutic utility unless there are clinical signs of infection, including drainage, presence of purulence in an aspirate, or erythema extending beyond the confines of the swelling. This evidence-based practice will translate into cost savings and minimize health care resource utilization and health care spending. Third, in clinically determined aseptic OB, steroids can be safely injected after fluid aspiration if this is the preference of the treating physician without the fear of missing an infection.

Two patients had CPPD crystals on fluid aspirate analysis. As all patients were prescribed a short course of non-steroidal anti-inflammatory drugs along with ice application, this result did not change our treatment plan. The 2 patients with positive CPPD crystals had an uneventful course without a recurrence compared with the CPPD-negative cohort. None of the aspirates were positive for MSU crystals.

Although the findings from this study demonstrate the limited value of microbiologic and crystal analysis of fluid aspirate from OB, these results should not be extrapolated to all types of OB. We do believe that laboratory analysis of the OB aspirate has a role in certain clinical scenarios. Patients with past medical history of gout, acute OB with drainage, erythema, or moderate-severe elbow pain will benefit from fluid analysis to assist in diagnosis other than aseptic OB. Furthermore, if there is any suspicion of infection based on unusual clinical examination findings, aspiration and fluid analysis of OB are warranted.

Our study is not without limitations. We had a total of 28 bursal aspirations included in this analysis. Although this cohort is small, we performed a post hoc power analysis based on an alpha of 0.05 and an incidence of 20% for septic OB as previously reported Aaron et al.^1^ This analysis demonstrated that our study was sufficiently powered (1 − β > 0.8) for our conclusions. We used the standard accepted laboratory cutoff values for synovial fluid analysis to diagnose septic OB, although there is no consensus on the cutoff parameters for white cell count and differential cell count for aseptic vs. septic OB. To make sure that a diagnosis of septic OB is not missed, we followed all patients clinically for 3 months to demonstrate that they did not have signs of missed infection.

## Conclusion

Our study demonstrates limited clinical utility of routine microscopic analysis (cell count, microbiologic, and crystal analysis) of fluid aspirate from clinically diagnosed aseptic OB.

## Disclaimers:

Funding: No funding was disclosed by the authors.

Conflicts of interest: The authors, their immediate families, and any research foundation with which they are affiliated have not received any financial payments or other benefits from any commercial entity related to the subject of this article.
